# Dexamethasone Administration in Mice Leads to Less Body Weight Gain over Time, Lower Serum Glucose, and Higher Insulin Levels Independently of NRF2

**DOI:** 10.3390/antiox11010004

**Published:** 2021-12-21

**Authors:** Fotini Filippopoulou, George I. Habeos, Vagelis Rinotas, Antonia Sophocleous, Gerasimos P. Sykiotis, Eleni Douni, Dionysios V. Chartoumpekis

**Affiliations:** 1 Division of Endocrinology, Department of Internal Medicine, School of Medicine, University of Patras, 26504 Patras, Greece; fotinifilip09@gmail.com (F.F.); gchabaios@gmail.com (G.I.H.); 2 Laboratory of Genetics, Department of Biotechnology, Agricultural University of Athens, 11855 Athens, Greece; vagrino@gmail.com (V.R.); or douni@aua.gr (E.D.); 3 Institute for Bioinnovation, Biomedical Sciences Research Center “Alexander Fleming”, 16672 Vari, Greece; 4 Department of Life Sciences, School of Sciences, European University Cyprus, Nicosia 2404, Cyprus; A.Sophocleous@euc.ac.cy; 5 Service of Endocrinology, Diabetology and Metabolism, Lausanne University Hospital and University of Lausanne, 1011 Lausanne, Switzerland; gerasimos.sykiotis@chuv.ch

**Keywords:** KEAP1, diabetes, glucocorticoids, osteoporosis, insulin resistance, gluconeogenesis, antioxidants

## Abstract

Glucocorticoids are used widely on a long-term basis in autoimmune and inflammatory diseases. Their adverse effects include the development of hyperglycemia and osteoporosis, whose molecular mechanisms have been only partially studied in preclinical models. Both these glucocorticoid-induced pathologies have been shown to be mediated at least in part by oxidative stress. The transcription factor nuclear erythroid factor 2-like 2 (NRF2) is a central regulator of antioxidant and cytoprotective responses. Thus, we hypothesized that NRF2 may play a role in glucocorticoid-induced metabolic disease and osteoporosis. To this end, WT and *Nrf2* knockout (*Nrf2*KO) mice of both genders were treated with 2 mg/kg dexamethasone or vehicle 3 times per week for 13 weeks. Dexamethasone treatment led to less weight gain during the treatment period without affecting food consumption, as well as to lower glucose levels and high insulin levels compared to vehicle-treated mice. Dexamethasone also reduced cortical bone volume and density. All these effects of dexamethasone were similar between male and female mice, as well as between WT and *Nrf2*KO mice. Hepatic NRF2 signaling and gluconeogenic gene expression were not affected by dexamethasone. A 2-day dexamethasone treatment was also sufficient to increase insulin levels without affecting body weight and glucose levels. Hence, dexamethasone induces hyperinsulinemia, which potentially leads to decreased glucose levels, as well as osteoporosis, both independently of NRF2.

## 1. Introduction

Chronic glucocorticoid administration is widely used in a variety of clinical settings, such as autoimmune and inflammatory diseases, with the purpose of symptomatic relief and prevention of disease progression. Approximately 1% of all adults and 3% of adults older than 50 in the USA receive glucocorticoids [1]. However, such use is associated with several adverse effects that include diabetes [2] and osteoporosis [3], and whose severity depends on the dose and duration of glucocorticoid exposure. Diabetes has been linked to oxidative stress as increased reactive oxygen species (ROS) are found in the plasma and adipose tissue of diabetic subjects [4], and hyperglycemia itself has been shown to lead to increased oxidative stress [5]. Glucocorticoids also have the potential to increase ROS generation and cause insulin resistance [6] and osteoporosis [7]. Nuclear erythroid factor 2-like 2 (NRF2) is a cytoprotective transcription factor that is activated upon exposure to ROS and upregulates the expression of antioxidant enzymes [8]. Its cytoplasmic inhibitor Kelch-like ECH-associated protein 1 (KEAP1) is rich in cysteines that react with ROS and with pharmacological activators of the NRF2 pathway, such as sulforaphane [9] and CDDO-Im [10]. This causes allosteric changes in KEAP1 that render it unable to bind more NRF2 molecules. Thus, de novo transcribed and translated NRF2 enters the nucleus and binds to specific DNA sequences called AREs (antioxidant response elements) in the regulatory regions of its target genes, thereby inducing their expression [11]. Activation of NRF2 has been shown to protect β cells against oxidative and nitrosative damage in mouse models of metabolic disease [12,13] and to prevent diet-induced obesity and type 2 diabetes [14,15].

In view of the antioxidant roles of NRF2 and the potential implication of dexamethasone-induced ROS in the pathogenesis of insulin resistance and osteoporosis, we hypothesized that NRF2 might play a protective role against the deleterious effects of dexamethasone on metabolism and bone. No studies have examined the role of NRF2 in the effects of glucocorticoids regarding diabetes and osteoporosis, and the few relevant prior studies that employed rodents treated with glucocorticoids used only male mice and were mostly focused on other outcomes than glucose metabolism and osteoporosis. Specifically, obese male mice showed lower glucose tolerance [16,17], lean male mice showed lower glucose levels [18,19], and both models gained less weight after various doses and duration of glucocorticoid treatments [16,17,18,19]. Herein, we performed a comprehensive investigation of the effects of glucocorticoids in mice by treating animals chronically with dexamethasone, a long-acting glucocorticoid. To examine the potential involvement of NRF2 in the glucocorticoid-induced phenotypes, both wild-type and *Nrf2*KO mice were used. In the metabolic studies, mice of both genders were included to address the possibility of a sexually dimorphic response to glucocorticoids. It was found that dexamethasone led to lower glucose levels and less weight gain in an NRF2- and gender-independent manner, as well as to reduced cortical bone volume and thickness in male mice in an NRF2-independent manner.

## 2. Materials and Methods

### 2.1. Mouse Model

All mouse experiments were performed at the University of Patras School of Medicine Animal Facility. The animal experiment protocol was approved by the relevant committee in the prefecture of Achaia in Patras, Greece and was given the number of approval 187574/630. The mice were housed at 22 °C and 50% humidity with a 12:12-h light-dark cycle with ad libitum access to water and food (standard diet 4RF21, Mucedola S.R.L., Settimo Milanese, Italy). All mice were in the C57BL/6J background. *Nrf2^−/−^* mice (*Nrf2*KO) were originally developed by Prof. M. Yamamoto [20] and were obtained from RIKEN BRC (Tsukuba, Japan). Wild-type (WT) and *Nrf2*KO mice were generated by mating *Nrf2^+/−^* mice and the offspring were genotyped as previously described [21] using the following primers: primer 1: 5′-TGGACGGGACTATTGAAGGCTG-3′, primer 2: 5′-GCGGATTGACCGTAATGGGATAGG-3′, primer 3: 5′-GCACTCGCGAGCTCCTCCATTTCCGAGTC-3′. Thermal cycling conditions were step 1: 95 °C for 180 s; step 2: 95 °C for 30 s; step 3: 70 °C for 30 s; step 4: 72 °C for 30 s; go to step 2 34 times; step 5: 72 °C for 120 s.

### 2.2. Dexamethasone Treatment of Mice

Three-month-old WT and *Nrf2*KO mice were randomized into groups based on gender, treatment and genotype, and were injected intraperitoneally 3 times per week (Monday-Wednesday-Friday) with 2 mg/kg pharmaceutical-grade dexamethasone (Dexaton^®^, Vianex SA, Athens, Greece) or vehicle (0.9% NaCl) for 13 weeks. Three to four mice were housed per cage. For measurement of food consumption, mice were single housed in metabolic cages during the 4th week of the treatment. At the end of the 13th week of treatment, mice were sacrificed after 4-h fasting. For the short-term experiments, WT male mice were administered 2 mg/kg dexamethasone or vehicle for two consecutive days before IPGTT (*n* = 5 per treatment) and another group (*n* = 4) was used for liver tissue collection. Hence, the following groups of mice were formed: WTc: wild-type control (vehicle-treated), *n* = 8 for male, *n* = 9 for female; KOc: *Nrf2* knockout control (vehicle-treated), *n* = 5 for male, *n* = 8 for female; WTdexa: wild-type dexamethasone-treated, *n* = 6 for male, *n* = 9 for female; KOdexa: *Nrf2* knockout dexamethasone-treated *n* = 5 for both male and female.

### 2.3. Intraperitoneal Glucose Tolerance Test (IPGTT) and Blood Chemistries Measurement

Mice were fasted for 8 h during a light cycle with free access to water before intraperitoneal administration of 1 g/kg D-glucose (MilliporeSigma, St Louis, MO, USA). Glucose was measured at the indicated time intervals with a Freestyle Precision Neo glucometer (Abbott Laboratories, Chicago, IL, USA). Insulin was measured by enzyme-linked immunosorbent assay (ELISA) using kit #10-1249-01 by Mercodia (Uppsala, Sweden). Serum was prepared by allowing blood to clot at room temperature for 30 min and by centrifugation at 2000× *g* for 15 min at 4 °C.

### 2.4. Micro-CT

Femurs were fixated in 10% formalin overnight at 4 °C and then washed and stored in PBS. The microarchitecture of the distal femurs from WTc, KOc, WTdexa, and KOdexa male mice was evaluated using a high-resolution SkyScan1172 microtomographic (microCT) imaging system (Bruker, Billerica, MA, USA). Images were acquired at 50 KeV, 100 µA with a 0.5 mm aluminum filter at a resolution of 6 μm. Three-dimensional reconstructions (8.8 mm cubic resolution) were generated using NRecon software (Bruker) and two-dimensional microCT images using DataViewer software (Brucker) as previously described [22,23]. Femoral trabecular geometry was assessed using 300 continuous CT slides (1800 µm) located at the trabecular area underneath the growth plate and femoral cortical geometry was assessed using 100 continuous CT slides (600 µm) located at the femoral midshaft as previously described [23]. The trabecular area of the distal femurs was assessed through measurement of the bone volume fraction (BV/TV, %), trabecular number (Tb.N, mm^−1^) and trabecular separation (Tb.Sp, mm). Cortical bone parameters included cortical bone volume fraction (Ct.BV/TV, %), average cortical width (Ct.Th, mm), and tissue mineral density (TMD, g/cm^3^).

### 2.5. RNA Preparation and Quantitative Real-Time PCR

Liver was excised from mice under ketamine/xylazine anesthesia 24 h after the last administration of dexamethasone and immersed in RNAlater solution (ThermoFisher Scientific, Pittsburgh, PA, USA) and preserved overnight at 4 °C and at −20 °C for longer-term storage. Total RNA was prepared by homogenizing the tissues in TRIzol reagent (ThermoFisher Scientific) following the manufacturer’s instructions and by further purification using the NucleoSpin RNA kit (Macherey-Nagel, Düren, Germany). Quantification of RNA was performed by photometry at 260 nm using the Multiskan Sky (ThermoFisher Scientific). The purity of RNA was estimated by calculating ratios of absorbance: 260 nm/280 nm and 260 nm/230 nm. RNA integrity was assessed by agarose gel electrophoresis. cDNA was synthesized using a PrimerScript RT Reagent Kit (Takara, Kusatsu, Japan). Real-time PCR was performed on a Step One Plus instrument (Applied Biosystems, Foster City, CA, USA) using SYBR Green KAPA SYBR FAST (Kapa Biosystems, Wilmington, MA, USA) in triplicate 20 μL reactions. PCR efficiency was calculated by standard curves, and the fold changes were calculated using the Pfaffl method [24]. The specificity of the amplified products was ensured by melting curves. Relative quantities were normalized to reference genes that were selected by employing the geNorm algorithm [25]. The geometric means of the relative quantities of *Rps29*, *Gusb*, and *Ppia* were used as a normalizer. The primer sequences are mostly derived from Primer Bank [26] and are shown in Table 1.

### 2.6. Statistics

Data are expressed as means ± SD. Student’s t-test and one-way ANOVA followed by Tukey’s multiple comparison test were used to compare two or more than two groups, respectively. GraphPad Prism 9 (GraphPad Software, La Jolla, CA, USA) was used for the statistical analysis and for the generation of graphs. *p* < 0.05 was considered significant.

## 3. Results

### 3.1. Chronic Dexamethasone Treatment Led to Less Weight Gain over Time in Male and Female Mice in an Nrf2-Independent Manner

**Figure 1:**
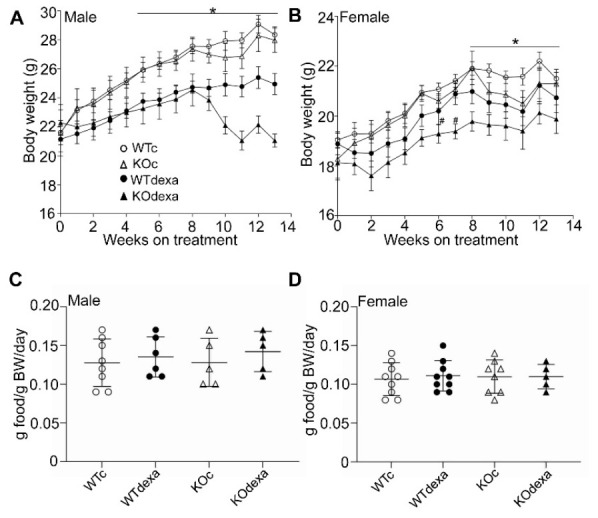
Body weights (BWs) and food consumption. (**A**) Male and (**B**) female WT or *Nrf2*KO mice body weights plotted against time during 13 weeks of treatment with dexamethasone. * *p* < 0.05 for dexamethasone-treated mice of both genotypes versus vehicle-treated mice from week 5 and week 8 of treatment onwards. # *p* < 0.05 of *Nrf2*KO dexamethasone-treated mice versus vehicle-treated. WTc: wild-type control (vehicle-treated); KOc: *Nrf2* knockout control (vehicle-treated); WTdexa: wild-type dexamethasone-treated; KOdexa: *Nrf2* knockout dexamethasone-treated. (**C**). Male and (**D**) female WT and *Nrf2*KO mice food consumption per BW per day.

Three-month-old mice were treated with vehicle or dexamethasone 3 times per week for 13 weeks. WT and *Nrf2*KO mice treated with vehicle had similar body weights at baseline and gained weight as expected over the ensuing 13 weeks, without differences between the two genotypes (Figure 1A,B). Dexamethasone-treated mice of both genders gained less weight than their vehicle-treated counterparts irrespectively of their genotype (Figure 1A,B) except for male *Nrf2*KO mice, which tended to have a lower weight compared to WT after 9 weeks of treatment (Figure 1A). No difference was observed in food consumption between dexamethasone- and vehicle-treated mice or between the two genotypes (Figure 1C,D).

### 3.2. Dexamethasone-Treated Mice Showed Lower Baseline Blood Glucose Levels and Better Glucose Tolerance along with Higher Insulin Levels Irrespectively of Genotype

**Figure 2:**
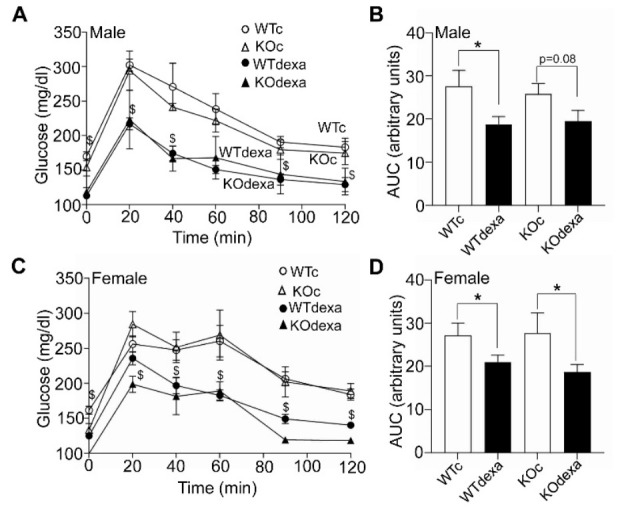
Intraperitoneal glucose tolerance tests (IPGTTs). (**A**) IPGTT in male WT and *Nrf2*KO mice treated with vehicle or dexamethasone for 12 weeks. (**B**) Area under the curve (AUC) of the IPGTT of male mice. (**C**) IPGTT in female WT and *Nrf2*KO mice treated with vehicle or dexamethasone for 12 weeks. (**D**) Area under the curve (AUC) of the IPGTT of female mice. * *p* < 0.05 of the indicated comparison, $ *p* < 0.05 of the dexamethasone-treated mice (either WT or *Nrf2*KO) versus the corresponding vehicle-treated mice of the same genotype.

At the beginning of the 13th week of dexamethasone treatment, mice underwent a glucose tolerance test. Baseline blood glucose levels were lower in dexamethasone-treated mice of both genders and genotypes (Figure 2A,C). No differences in glucose were observed between genotypes (Figure 2A,C). After glucose challenge, the dexamethasone-treated mice had lower levels of glucose at most time-points of the IPGTT (Figure 2A,C). This is also evident from the IPGTT area under the curve (AUC), with the dexamethasone-treated mice of both genders having lower AUC values compared to respective vehicle-treated mice (Figure 2B,D). No differences in AUC were observed between genotypes in both sexes (Figure 2B,D).

**Figure 3:**
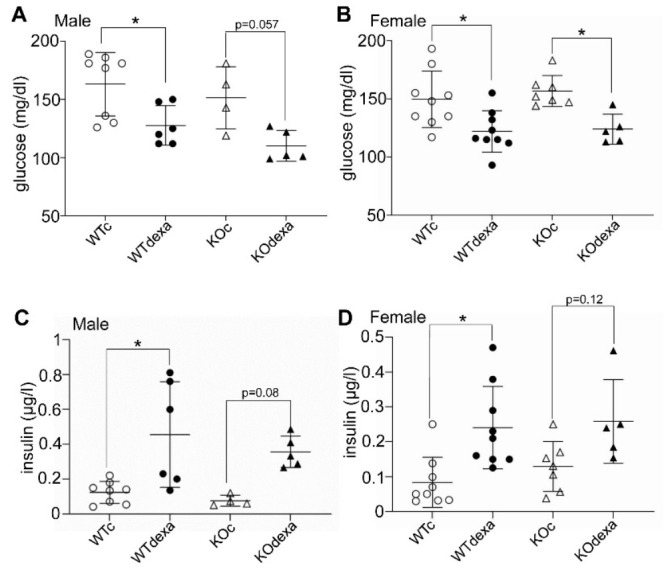
Serum levels of glucose in male (**A**) and female (**B**) mice, and insulin in male (**C**) and female (**D**) WT or *Nrf2*KO mice after 13 weeks of treatment with vehicle or dexamethasone. Blood was drawn after 4 h of fasting. * *p* < 0.05 of the indicated comparison. WTc: wild-type control (vehicle-treated); KOc: *Nrf2* knockout control (vehicle-treated); WTdexa: wild-type dexamethasone-treated; KOdexa: *Nrf2* knockout dexamethasone-treated.

In both genotypes and sexes, when mice were sacrificed at the end of the experiment, the baseline glucose levels of the dexamethasone-treated mice were lower than those of the vehicle-treated ones (Figure 3A,B), whereas their insulin levels were higher (Figure 3C,D).

### 3.3. Dexamethasone Treatment Decreased Cortical Bone Volume in Male Mice Independently of Nrf2

**Figure 4:**
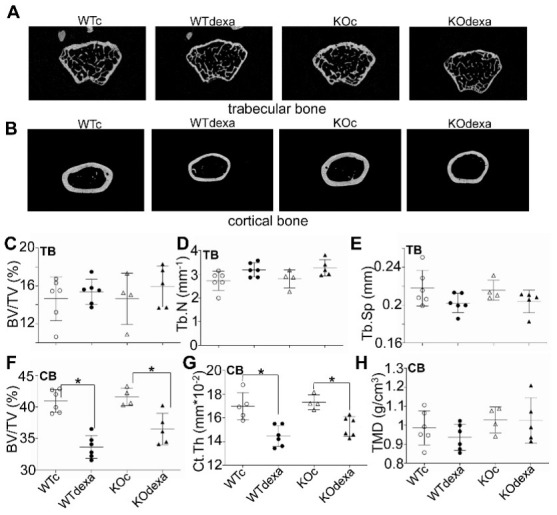
MicroCT analysis of distal femurs in male mice. Representative 2D longitudinal microCT images of (**A**) trabecular bone at the metaphyseal region in distal femurs and of (**B**) cortical bone at the diaphysis region of distal femurs from each each experimental group. Quantitative analysis of the trabecular bone in the metaphysis region of the distal femurs in male mice: (**C**) BV/TV (bone volume/total volume, %), (**D**) Tb.N (trabecular number per mm), (**E**) Tb.Sp (trabecular separation, mm). Quantitative analysis of midshaft cortical bone of femurs from each group with microCT: (**F**) BV/TV (bone volume/total volume, %), (**G**) Ct.Th (cortical thickness, mm), (**H**) TMD (Tissue mineral density, g/cm^3^). * *p* < 0.05 of the indicated comparison. WTc: wild-type control (vehicle-treated); KOc: *Nrf2* knockout control (vehicle-treated); WTdexa: wild-type dexamethasone-treated; KOdexa: *Nrf2* knockout dexamethasone-treated. TB: trabecular bone. CB: cortical bone.

The femurs of mice were scanned with microCT to assess any changes in bone morphometry in response to dexamethasone treatment in both WT and *Nrf2*KO mice. Due to the cost and complexity of these experiments, only male mice were studied. Both trabecular and cortical bone were examined (Figure 4). Dexamethasone treatment was found to reduce cortical bone volume (BV/TV, %) (Figure 4F) and thickness (Figure 4B,G) in both WT and *Nrf2*KO mice, without a difference between the two genotypes. Regarding trabecular bone, no significant difference was found in the various morphometric parameters (Figure 4C–E).

### 3.4. Chronic Dexamethasone Exposure Does Not Affect the mRNA Expression Levels of Key Gluconeogenic Enzymes and NRF2 Signaling-Related Genes in Liver

**Figure 5:**
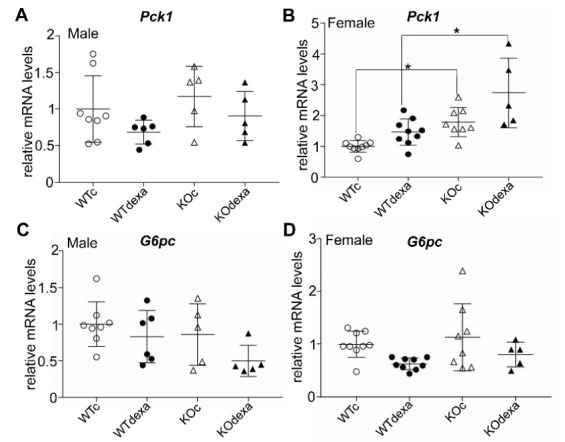
Relative mRNA expression of *Pck1* and *G6pc* in liver of male (**A**,**C**, respectively) and female (**B**,**D** respectively) WT or *Nrf2*KO mice treated with vehicle or dexamethasone. * *p* < 0.05 of the indicated comparison. WTc: wild-type control (vehicle-treated); KOc: *Nrf2* knockout control (vehicle-treated); WTdexa: wild-type dexamethasone-treated; KOdexa: *Nrf2* knockout dexamethasone treated.

Analysis of the mRNA expression of the rate-limiting gluconeogenic enzymes *Pck1* and *G6pc* did not reveal any difference between vehicle- and dexamethasone-treated mice in both genotypes and sexes (Figure 5). Female *Nrf2*KO mice had ~1.5-fold higher levels of *Pck1* in both vehicle- and dexamethasone-treated groups (Figure 5B).

**Figure 6:**
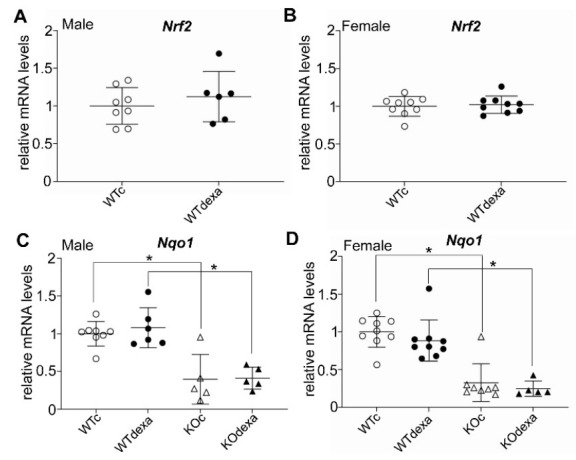
Relative mRNA expression of *Nrf2* and its prototypical target gene Nqo1 in liver of male (**A**,**C**, respectively) and female (**B**,**D**, respectively) WT or *Nrf2*KO mice treated with vehicle or dexamethasone. * *p* < 0.05 of the indicated comparison. WTc: wild-type control (vehicle-treated); KOc: *Nrf2* knockout control (vehicle-treated); WTdexa: wild-type dexamethasone-treated; KOdexa: *Nrf2* knockout dexamethasone-treated.

Chronic exposure to dexamethasone did not affect the mRNA levels of *Nrf2* in both genders (Figure 6A,B), while the mRNA levels of the *Nrf2* prototypical target gene *Nqo1* were not affected by dexamethasone treatment in either gender (Figure 6C,D). As expected, levels of *Nqo1* mRNA were significantly lower in the *Nrf2*KO mice compared to their WT counterparts (Figure 6C,D).

### 3.5. Acute Dexamethasone Treatment Caused Hyperinsulinemia without Affecting Gluconeogenic Gene Expression or Nrf2 Signaling

**Figure 7:**
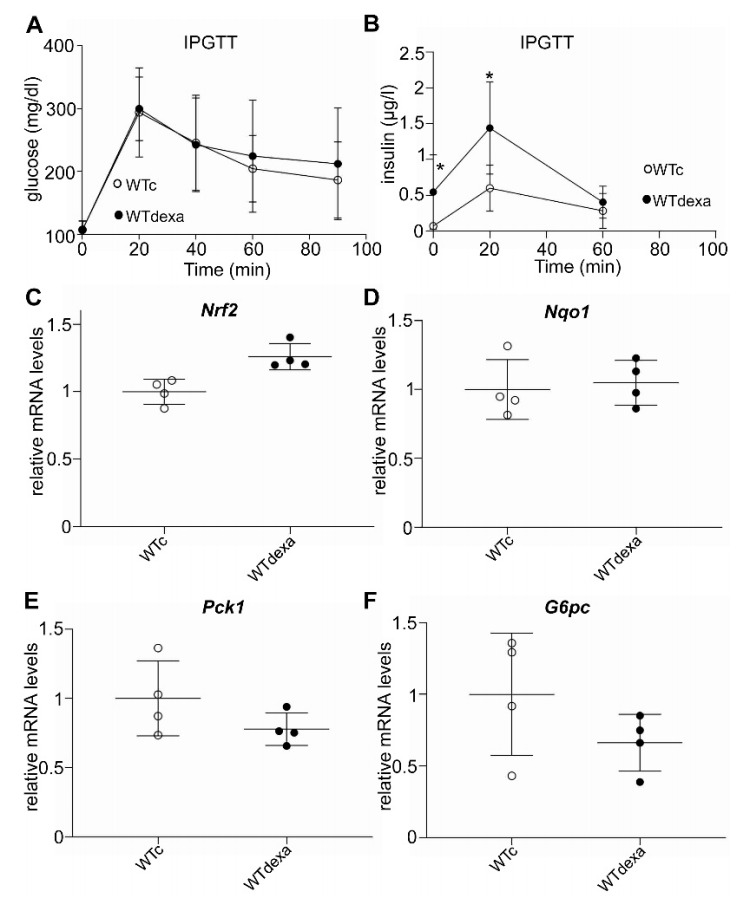
Intraperitoneal glucose tolerance test (IPGTT) (**A**) and measurement of insulin levels at certain timepoints during the test (**B**) in WT mice treated short term (2 consecutive days) with vehicle or dexamethasone. Relative mRNA expression of *Nrf2* (**C**), *Nqo1* (**D**), *Pck1* (**E**), and *G6pc* (**F**) in livers of WT mice treated short term with vehicle or dexamethasone. * *p* < 0.05 compared to vehicle-treated. WTc: wild-type control (vehicle-treated); WTdexa: wild-type dexamethasone-treated.

Because long-term treatment with dexamethasone led to less weight gain, a short-term dexamethasone treatment experiment was performed to examine whether the increased insulin levels are weight independent. Female WT mice were treated with 2 mg/kg dexamethasone or vehicle every 24 h for 2 consecutive days, and an IPGTT was performed 24 h after the last dose. Baseline glucose levels were not different between vehicle-treated and dexamethasone-treated mice (Figure 7A), even though dexamethasone-treated mice had higher insulin levels (Figure 7B) that remained elevated up to the 20′ time point of the IPGTT (Figure 7B). Glucose levels were not different between groups during the IPGTT (Figure 7A).

Given that a short-term dexamethasone treatment has been shown to attenuate genetically and pharmacologically activated NRF2 signaling in the liver [27], we investigated whether this also applies to the baseline NRF2 signaling activation status. To this end, the liver mRNA levels of *Nrf2*, *Nqo1*, and the key gluconeogenic genes *Pck1* and *G6pc* were assessed after acute dexamethasone treatment. Similar to what was observed after the long-term dexamethasone treatment (Figure 6), no attenuation of NRF2 signaling was found after short-term dexamethasone treatment (Figure 7C,D). No differences were found in *Pck1* and *G6pc* mRNA expression levels either (Figure 7E,F).

## 4. Discussion

In the present study, we examined the effect of dexamethasone on glucose metabolism and osteoporosis under a standard diet in two genotypes (WT and *Nrf2*KO). We showed that chronic dexamethasone treatment in mice under a standard diet led to less body weight gain over time and, surprisingly, to lower glucose levels. These observations did not show sexual dimorphism and were independent of the presence of NRF2. The decreased glucose levels after dexamethasone exposure contradicts what is regularly expected in human patients treated chronically with glucocorticoids who develop hyperglycemia. Glucocorticoid-induced hyperglycemia is also common in mouse models of insulin resistance, such as high-fat diet-induced obesity [16] and *db*/*db* mice [17]. In our study, young (3-month-old) mice were used and were fed a standard diet, which per se is not expected to induce insulin resistance. Although the lower glucose levels observed in our glucocorticoid-treated model were seemingly unusual, previous studies conducted with objectives other than glucose metabolism reported similar results. Specifically, mice treated for 1 week with dexamethasone tended to have lower glucose levels [19]. In another study, 5 daily injections of dexamethasone also led to lower blood glucose levels after a 6-h fasting [18]. In addition, a 7-day dexamethasone treatment of rhesus macaques also resulted in lower postprandial glucose levels [28]. Lastly, treatment of mice on a standard diet with 2 mg/kg dexamethasone every other day led to lower glucose levels and ameliorated glucose tolerance [29], similar to what was seen in our study (Figure 2 and Figure 3).

We propose that the observed lower glucose levels in our model can be attributed at least partially to the higher insulin levels after dexamethasone treatment. It is known that both acute and chronic administration of glucocorticoids induce insulin resistance in skeletal muscle [30,31] and are accompanied by high insulin levels [32,33]. The increased insulin levels in our study were observed in both the short-term (2 days) and long-term (13 weeks) dexamethasone treatments, which supports the notion that the observed lower glucose levels were not related to the lower body weight in the chronically treated mice. Hence, one plausible explanation is that dexamethasone induces hyperinsulinemia that leads to lower glucose levels. This has also been reported in a study of young adult rhesus macaques that received dexamethasone for 7 consecutive days and showed improved glucose tolerance after feeding, accompanied by marked hyperinsulinemia [28]. Moreover, mice injected with dexamethasone for 5 days showed decreased glucose and higher insulin levels after 6 h of fasting compared to the vehicle-treated mice [18]. Consistent with these observations, pancreatic islets isolated from rats treated with dexamethasone for 5 days secreted insulin at lower levels of glucose compared to control islets [34]. In conclusion, the lower glucose levels after dexamethasone treatment could be attributed to hyperinsulinemia that may be due to both increased insulin resistance in muscle and increased sensitivity of pancreatic β cells to lower glucose levels.

The lower weight gain in dexamethasone-treated mice is another factor that could lead to lower glucose levels. Dexamethasone-treated mice of both sexes showed lower weight gain over time compared to the control and the male mice tended to show weight loss towards the end of the treatment (Figure 1A,B). This observation is consistent with several prior studies with variable experimental designs. Specifically, mice on a standard or high-fat diet gained less weight when they were treated with dexamethasone for 12 [16] or 7 weeks [35]. Five [18] or 7 days [19] of dexamethasone treatment of mice on a standard diet also resulted in less weight gain. Rats treated with 2.5 mg/kg of dexamethasone for 14 weeks or 0.5 mg/kg for 10 days also gained significantly less weight [36,37]. Treatment with 100 mg/kg hydrocortisone for 20 days also reduced weight gain in rats [38]. The lower body weight gain after glucocorticoid treatment could be attributed to lower lean mass, as has been reported in other studies [29,37]. Although lean body mass and fat mass were not measured in our study, cortical bone volume was significantly reduced after dexamethasone treatment (Figure 4J,K) and that could at least partially contribute to the observed weight differences. The lower weight gain after dexamethasone treatment could not be attributed to lower food consumption, as the latter was similar between the vehicle- and dexamethasone-treated groups (Figure 1C,D).

It is known that dexamethasone reduces bone mass [39,40]. In humans, glucocorticoid-induced osteoporosis is a clinical problem that is often encountered due to the prevalent use of these drugs. Age older than 55 years and female sex are risk factors for osteoporosis upon glucocorticoid use [41]. Moreover, there are relatively few data on the role of NRF2 in bone metabolism; prior studies have shown that the absence of NRF2 negatively affects bone mass in ovariectomized mice [42] or in mice exposed to arsenic [43]. No studies have examined the role of NRF2 in dexamethasone-induced osteoporosis. Herein, we showed that dexamethasone led to reduced cortical bone volume and thickness in male mice, and that this effect was not dependent on the presence of NRF2 (Figure 4J–L). Recently, WT and *Nrf2*KO mice that were subjected to space travel, another stimulus for loss of bone mass, were found to lose bone density equally [44]. Notwithstanding these observations, it remains interesting to investigate whether activation of NRF2 by genetic or pharmacologic factors might affect dexamethasone-induced osteoporosis.

It is known that NRF2 pathway activation prevents diet-induced obesity and type 2 diabetes in high-fat diet-fed mice and represses gluconeogenesis and lipogenesis [14,15]. Under a standard diet, no such effects of NRF2 are observed, indicating that the impact of NRF2 signaling might be important only under conditions that increase oxidative stress. There are no studies regarding the potential therapeutic potential of NRF2 in dexamethasone-induced metabolic disturbances. The lack of NRF2 involvement documented in our study might be explained by the fact that our experimental mice were not under oxidative stress at baseline due to the absence of obesity or diabetes. It has also been shown that dexamethasone treatment attenuates the activation of NRF2 signaling by chemical inducers of NRF2 (CDDO-Im, diethyl-maleate) in vitro and in vivo [27]. In our study, short- and long-term dexamethasone treatment did not affect the mRNA transcript levels of *Nrf2* or its prototypical target gene *Nqo1* at baseline (Figure 6 and Figure 7C,D). Similarly, dexamethasone did not affect the mRNA levels of the gluconeogenic genes *Pck1* and *G6pc* in our model (Figure 5 and Figure 7) probably due to hyperinsulinemia-induced repression of gluconeogenesis (Figure 3 and Figure 7). Even though a lack of NRF2 in the context of obesity leads to increased transcription of gluconeogenic genes [45,46] and activation of NRF2 leads to their repression [14,15], gluconeogenic gene expression was not significantly affected by the absence of NRF2 after short- and long-term dexamethasone treatment (Figure 3 and Figure 7). In summary, based on the present findings and prior literature, further studies are warranted to examine the role of NRF2 in dexamethasone treatment using models of obesity or diabetes with gain or loss of NRF2 signaling. Such studies are relevant and timely in view of the widespread use of dexamethasone in obese and diabetic patients hospitalized for COVID-19 infection.

## 5. Conclusions

- Dexamethasone induced hyperinsulinemia with concomitant low glucose levels independently of NRF2.
- Acute or chronic dexamethasone treatment did not affect NRF2 signaling.
- All the aforementioned effects of dexamethasone were independent of gender.
- Dexamethasone-induced osteoporosis in male mice was independent of NRF2.

## Figures and Tables

**Table 1:** Primers used for real-time PCR.

Gene	Gene NCBI ID	Forward Primer	Reverse Primer
*G6pc*	14377	CTAGCTTTGATCTGGTTGTCAG	GTTGAACCAGTCTCCGACCA
*Gusb*	110006	ACTGACACCTCCATGTATCCCAAG	CAGTAGGTCACCAGCCCGATG
*Nrf2*	18024	CTTTAGTCAGCGACAGAAGGAC	AGGCATTCTTGTTTGGGAATGTG
*Nqo1*	18104	CATTCTGAAAGGCTGGTTTGA	CTAGCTTTGATCTGGTTGTCAG
*Pck1*	18534	CTGCATAACGGTCTGGACTTC	CAGCAACTGCCCGTACTCC
*Ppia*	268373	CAGACGCCACTGGTCGCTTT	TGTCTTTGGAACTTTGTCTGCAA
*Rps29*	20090	TCTACTGGAGTCACCCACGGAA	GGAAGCACTGGCGGCACA

## Data Availability

All of the data is contained within the article.
